# Effects of T2DM on cancer progression: pivotal precipitating factors and underlying mechanisms

**DOI:** 10.3389/fendo.2024.1396022

**Published:** 2024-09-03

**Authors:** Yu-Yuan Zhang, Yong-Jiang Li, Chun-Dong Xue, Shen Li, Zheng-Nan Gao, Kai-Rong Qin

**Affiliations:** ^1^ Institute of Cardio-Cerebrovascular Medicine, Central Hospital of Dalian University of Technology, Dalian, Liaoning, China; ^2^ School of Biomedical Engineering, Faculty of Medicine, Dalian University of Technology, Dalian, Liaoning, China

**Keywords:** T2DM, cancer, molecular mechanism, hyperinsulinemia, hyperglycemia, hyperlipidemia

## Abstract

Type 2 diabetes mellitus (T2DM) is a chronic metabolic disorder affecting people worldwide. It is characterized by several key features, including hyperinsulinemia, hyperglycemia, hyperlipidemia, and dysbiosis. Epidemiologic studies have shown that T2DM is closely associated with the development and progression of cancer. T2DM-related hyperinsulinemia, hyperglycemia, and hyperlipidemia contribute to cancer progression through complex signaling pathways. These factors increase drug resistance, apoptosis resistance, and the migration, invasion, and proliferation of cancer cells. Here, we will focus on the role of hyperinsulinemia, hyperglycemia, and hyperlipidemia associated with T2DM in cancer development. Additionally, we will elucidate the potential molecular mechanisms underlying their effects on cancer progression. We aim to identify potential therapeutic targets for T2DM-related malignancies and explore relevant directions for future investigation.

## 1 Introduction

Type 2 diabetes mellitus (T2DM) is a chronic metabolic disorder primarily characterized by insulin resistance and relative insulin deficiency (1). This condition leads to persistent hyperglycemia (2–4). T2DM accounts for approximately 90% of all diabetes cases worldwide. It is often associated with various systemic complications, including cardiovascular disease, nephropathy, neuropathy, and retinopathy (3, 4). Hyperglycemia is a hallmark of T2DM, resulting from the body’s inability to efficiently use glucose due to impaired insulin action (5–10). This chronic elevation of blood glucose levels is a key factor in the development of T2DM-related complications (9–11).

In addition to hyperglycemia, T2DM is often associated with hyperinsulinemia. This condition is an early compensatory response to insulin resistance, where the pancreas produces more insulin to overcome reduced insulin sensitivity (12, 13). Over time, hyperinsulinemia can lead to pancreatic β-cell dysfunction, further exacerbating hyperglycemia. T2DM is also linked with dyslipidemia, characterized by elevated levels of triglycerides and low-density lipoprotein (LDL) cholesterol, along with decreased levels of high-density lipoprotein (HDL) cholesterol (14–16). This lipid imbalance further contributes to the increased risk of cardiovascular disease in T2DM patients.

**Figure 1:**
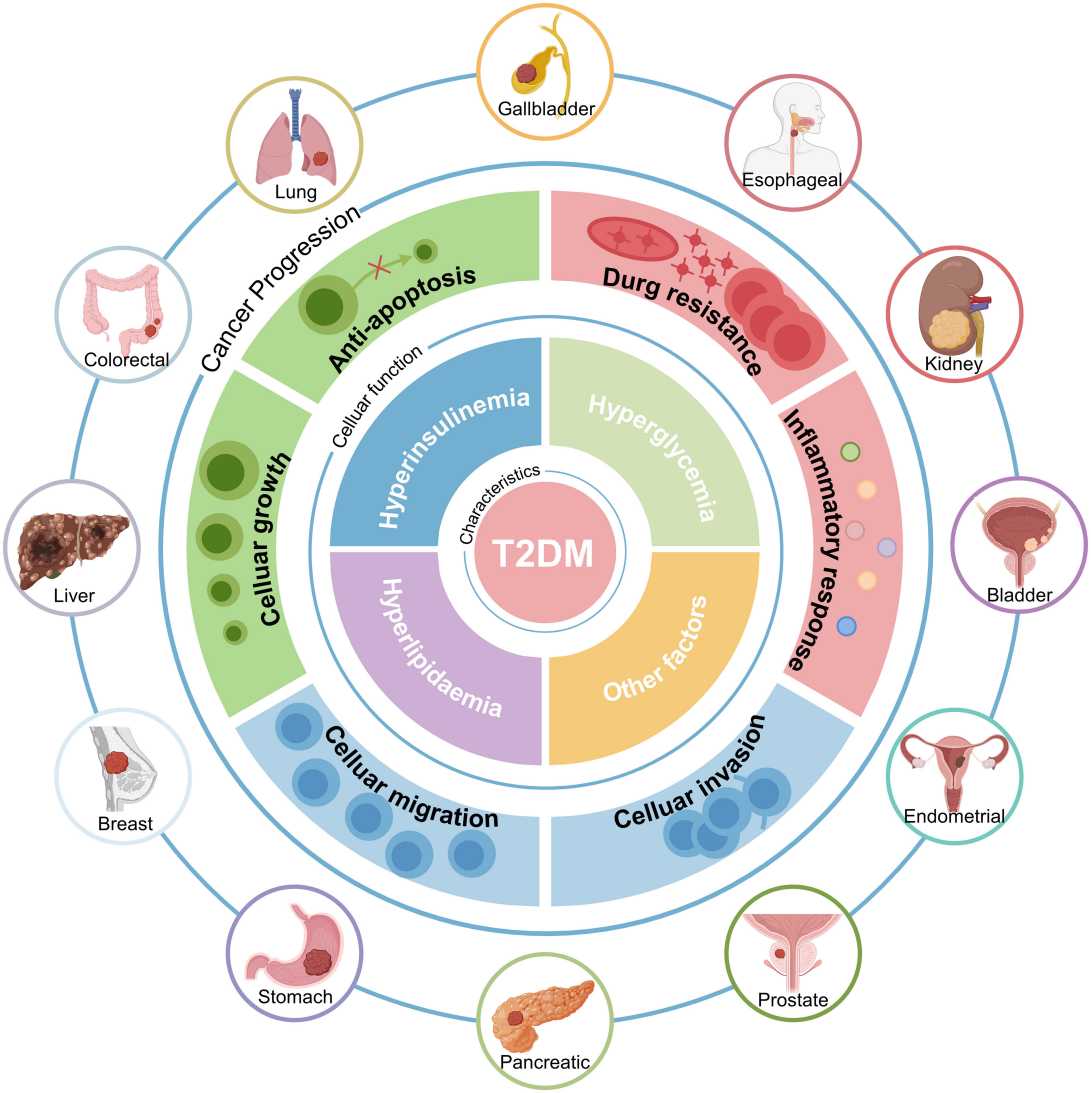
Patients with T2DM have a Higher Cancer Risk. The increased risk of cancer in people with T2DM is intricately associated with comorbid hyperinsulinemia, hyperglycemia, hyperlipidemia, and other factors. T2DM, Type 2 diabetes mellitus.

Epidemiologic studies consistently show that individuals with T2DM have an increased risk of developing several malignancies (17–20). These include lung, colorectal, liver, breast, gastric, and pancreatic cancers (21). The pathological features of T2DM, particularly hyperinsulinemia, hyperglycemia, and hyperlipidemia, are believed to contribute to this increased cancer risk (**Figure 1**). Hyperglycemia promotes cancer cell proliferation by providing a readily available source of glucose, fueling rapid cell division (22, 23). Hyperinsulinemia promotes tumor growth and progression through its mitogenic effects by activating insulin and insulin-like growth factor (IGF) signaling pathways (24, 25). Dyslipidemia contributes to chronic inflammation and oxidative stress, creating a pro-tumorigenic environment (26–28).

Despite these associations, the precise molecular mechanisms linking T2DM-related features to cancer progression remain incompletely understood. In this review, we will provide an overview of the epidemiologic associations between T2DM and cancer. We will also explore the potential molecular mechanisms involved. Specifically, we will examine how hyperglycemia, hyperinsulinemia, and hyperlipidemia may accelerate cancer development and progression in patients with T2DM.

## 2 Epidemiological association between T2DM-related features and cancer

In recent years, clinical researchers have conducted extensive epidemiological studies to elucidate the relationship between these two diseases (29). The majority of the findings from these studies consistently show a significantly increased risk of cancer among individuals with T2DM. To gain a clearer understanding of the association between T2DM and cancer, this section compiles and summarizes epidemiological research on the three key features of T2DM: hyperinsulinemia, hyperglycemia, and hyperlipidemia in relation to cancer.

Numerous epidemiological studies have consistently shown that the increased risk of cancer among people with T2DM is significantly influenced by hyperinsulinemia. Researchers have discovered that hyperinsulinemia increases the risk of colorectal, breast, endometrial, hepatocellular, and prostate cancers through assessments of the relationship between the empirical dietary index for hyperinsulinemia (EDIH) and specific cancer risks (30–34). Furthermore, hyperinsulinemia increases the likelihood of cancer recurrence in individuals with prostate cancer (PCa), breast cancer, and curative hepatocellular carcinoma (HCC) (35–37). While being unrelated to survival rates in individuals with late-stage colon cancer, a prospective investigation found that a higher EDIH associated with hyperinsulinemia may increase the incidence risk of colon cancer (38). Moreover, one of the contributing factors to hastening mortality in cancer patients is hyperinsulinemia (39). Specific research results have indicated that hyperinsulinemia increases the probability of dying from colorectal, gastroenteric, and hepatic malignancies by 1.51-fold, 1.61-fold, and 2.72-fold, respectively (40–42). It is noteworthy that hyperinsulinemia raises the risk of death among cancer patients regardless of whether individuals fall into the obese category (43). Opting for meals with a lower insulinemic potential may be a useful strategy to improve general health and prevent early death (44). The findings of the previous study demonstrate that hyperinsulinemia may independently influence cancer incidence and mortality rates.

While glycemic control has received significant attention in the context of T2DM, the importance of hyperglycemia as a key component in tumor growth is sometimes overlooked. Nevertheless, epidemiological research has established a connection between hyperglycemia and cancer incidence, progression, and mortality rates (7). For instance, in a study investigating the impact of blood sugar on cancer risk in women from the 1987–1992 ORDET cohort, conditional logistic regression was employed to determine the rate ratios (RR). The results indicated that women with blood sugar levels in the highest quartile had a significantly higher risk of breast cancer compared to those in the lowest quartile (RR 1.63; 95% CI: 1.14-2.32; trend *p*-value = 0.003) (45). Additionally, a recent systematic study sheds light on the major influence of diabetes and hyperglycemia on the occurrence of colon cancer (46). Although several cohort studies have emphasized the link between diabetes and cancer risk, only a limited number of studies have directly examined the underlying impact of high blood sugar on cancer risk (7). At present, clinical studies are the main focus of research on the relationship between hyperglycemia and cancer (47). Clinical research has demonstrated that elevated blood glucose is associated with poor clinical outcomes in patients with T2DM and cancer (5). Conversely, the prognosis for cancer in diabetic people improves when plasma glucose levels return to normal. This suggests that T2DM and cancer share common signaling pathways that hyperglycemia may activate. Therefore, regulating plasma glucose levels in people with cancer and T2DM may improve the prognosis for cancer.

Hyperlipidemia primarily develops as a result of dysregulated lipid metabolism. Lipids serve crucial functions in cellular signaling, chemical transport, and energy storage and they are essential components of cell membranes. An increasing body of evidence from recent studies indicates that abnormal lipid metabolism increases the risk of tumorigenesis, disease progression, and treatment resistance (28, 48). Excess lipid and cholesterol accumulation within cancer cells is stored in lipid droplets, and higher lipid droplet levels and stored cholesterol ester content are now considered indicators of cancer invasion (49). Furthermore, lipid metabolism reprogramming has emerged as a new factor in cancer development (28). Recent epidemiological studies have highlighted the critical role of lipid metabolism in the progression of various cancers, including breast cancer (50), thyroid cancer (51), prostate cancer (52), and ovarian cancer (53). Elevated serum cholesterol levels have been linked to an increased risk of colon, rectal, prostate, and testicular cancers (54). Interestingly, low cholesterol levels have occasionally been associated with cancer. A prospective study based on the Japan Public Health Center found a significant correlation between low cholesterol levels and the incidence of primary liver and stomach malignancies (55). Recent research has also linked low cholesterol to colon and lung malignancies (54, 56). However, due to the conflicting epidemiological results mentioned above, it is still unclear how cholesterol contributes to the development of cancer. According to some recent research, low cholesterol levels may be linked to impaired immune function, increasing one’s vulnerability to cancer (57). In contrast, low cholesterol levels may lower the risk of cancer by activating the nuclear factor-κB (NF-κB), which inhibits cell proliferation and promotes cell differentiation (58). Furthermore, extracellular vesicle proteins and lipids could serve as biomarkers for lung cancer diagnosis and prognosis, as well as therapeutic targets for drug development (59). While epidemiological research examines the impact of aberrant blood lipid levels on cancer, the heterogeneity of study results makes it challenging to establish causal correlations. Uncovering the specific pathways by which hyperlipidemia promotes cancer remains a critical task.

## 3 Hyperinsulinemia and cancer progression

**Figure 2:**
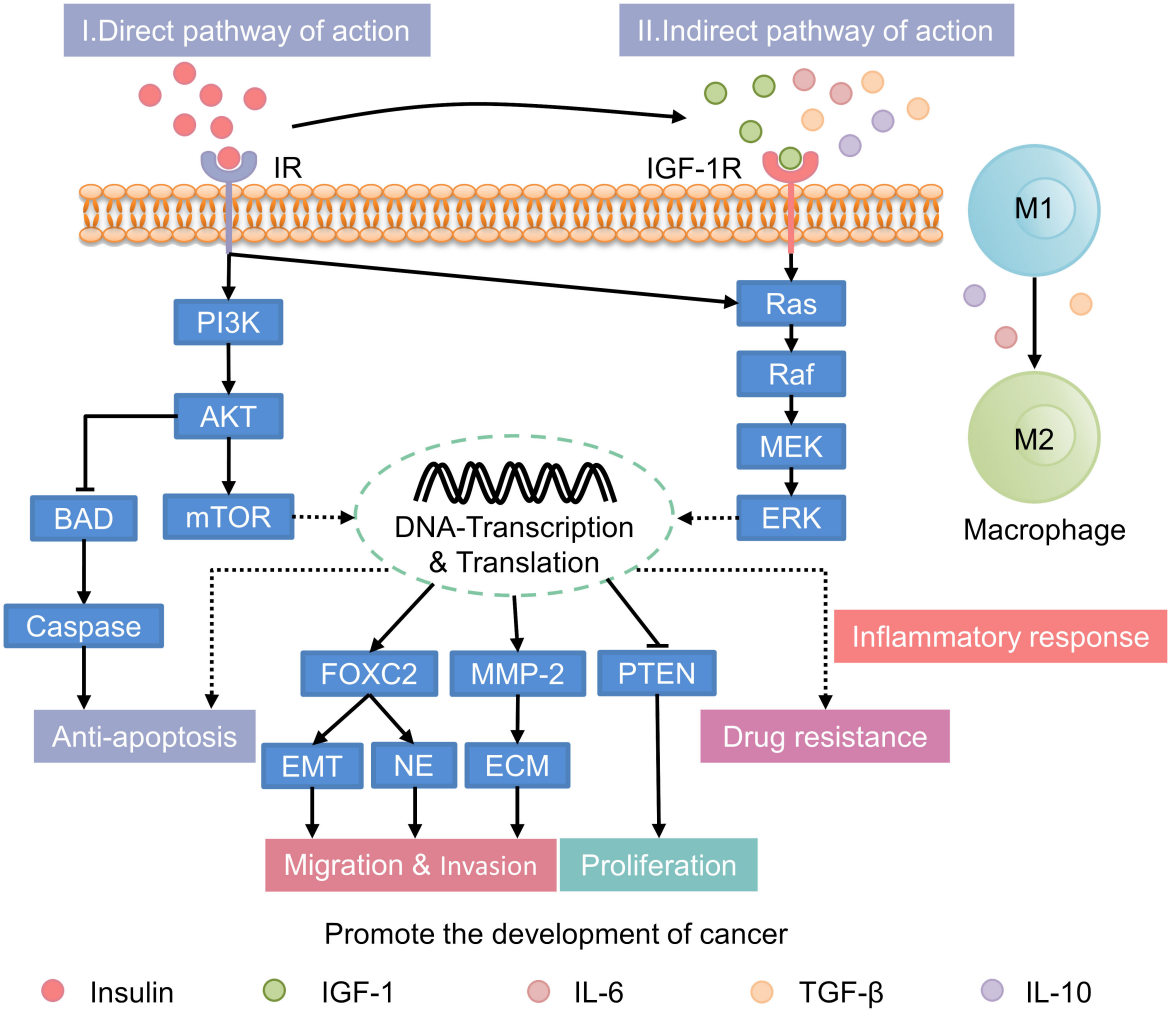
Mechanisms underlying the promotion of cancer progression by hyperinsulinemia. IR, insulin receptor; PI3K, phosphatidylinositol 3-kinase; AKT, protein kinase B; mTOR, mammalian target of rapamycin; BAD, Bcl2-associcated death protein; FOXC2, forkhead box protein C2; EMT, epithelial-to-mesenchymal transition; NE, neuroendocrine; MMP-2, matrix metalloproteinase-2; ECM, extracellular matrix; PTEN, phosphatase and tensin homolog deleted on chromosome 10; IGF-1, insulin-like growth factor-1; IL-6, interleukin-6; TGF-β, transforming growth factor-β; IL-10, interleukin-10; IGF-1R, IGF-1 receptor; MEK, mitogen-activated protein kinase; ERK, extracellular signal-regulated kinase.

In patients with T2DM, hyperinsulinemia is associated with an increased risk of cancer. It can also accelerate cancer progression through both direct and indirect mechanisms (**Figure 2**). These mechanisms involve the activation of key signaling pathways and the modulation of extracellular factors that influence tumor growth.

### 3.1 Direct mechanisms

Under normal conditions, insulin primarily regulates glucose metabolism with limited direct involvement in cancer progression. However, in individuals with hyperinsulinemia, the excessive activation of insulin signaling pathways, including the phosphatidylinositol 3-kinase/protein kinase B (PI3K/AKT), mTOR, and Ras/mitogen-activated protein kinase (MAPK) pathways, significantly enhances tumor growth (60–65). 1) PI3K/AKT Pathway: In hyperinsulinemic states, the PI3K/AKT pathway is persistently activated due to chronic insulin receptor (IR) stimulation, leading to enhanced tumor cell proliferation, migration, and survival (66). This pathway also inhibits apoptosis by downregulating pro-apoptotic proteins such as B-cell lymphoma-2 (Bcl-2)-associated death promoter and rapidly accelerated fibrosarcoma-1 (Raf-1), thereby promoting tumor cell survival (67). 2) mTOR Pathway: The mTOR pathway is activated by abnormal insulin signaling, enhancing synthetic metabolism, cell proliferation, and inhibiting autophagy (68). This contributes to tumor growth by facilitating the accumulation of biomolecules essential for cancer cell proliferation. 3) Ras/MAPK Pathway: Hyperinsulinemia also activates the Ras/MAPK pathway, which regulates cell division, migration, and proliferation (62). This pathway is particularly important in promoting the aggressive spread of tumors and is implicated in various cancers, including prostate and endometrial cancers (69).

### 3.2 Indirect mechanisms

Normally, systemic factors are balanced to regulate cell growth. In hyperinsulinemia, elevated insulin levels indirectly promote tumorigenesis. This occurs through alterations in the insulin-like growth factor-1 (IGF-1) and sex hormone-binding globulin (SHBG) system (13, 65). Insulin increases IGF-1 synthesis in the liver and upregulating growth hormone receptors. This leads to elevated IGF-1 levels in the bloodstream (70–72). In hyperinsulinemic states, these elevated IGF-1 levels result in enhanced proliferation, migration, and anti-apoptosis in HCC cell lines, such as SK-Hep1 and HepG2. Additionally, there is increased resistance to sorafenib. These effects are mediated by the regulation of the PI3K/AKT and RAS/Raf/ERK signaling pathways (24). In addition to increasing cancer risk through IGF-1, insulin also influences carcinogenesis and progression by lowering sex hormone-binding globulin (SHBG) levels (73). Normally, sex hormone levels are tightly regulated to maintain physiological balance. However, during conditions like menopause or hormonal imbalances, elevated sex hormones can increase the risk of certain cancers. For example, prolonged exposure to high estrogen levels is closely linked to the development of breast and endometrial cancers. In individuals with hyperinsulinemia, elevated insulin levels reduce SHBG. Lower SHBG levels lead to higher circulating levels of free estrogen and testosterone. This increase in active sex hormones may accelerating the onset and progression of hormone-related cancers, such as endometrial and breast cancers (74). Notably, recent research suggests a close relationship between sex hormones, such as estrogen and testosterone, and the expression of the insulin-degrading enzyme (IDE). High levels of these hormones can enhance the negative feedback of IDE on itself. Additionally, elevated hormone levels may increase the risk of hormone-dependent malignancies (75).

### 3.3 Inflammation and immune modulation

The inflammatory milieu associated with hyperinsulinemia is distinct from that of normal individuals, contributing to a more pro-tumorigenic environment. Persistent high levels of insulin can promote generation of an inflammation environment, which plays a key role in tumor initiation and progression (12). Studies have shown that hyperinsulinemic patients secrete more pro-inflammatory cytokines, including interleukin-6 (IL-6) and tumor necrosis factor-alpha (TNF-α), compared to normal individuals. Under inflammatory stimuli such as transforming growth factor-β (TGF-β), IL-6, and interleukin-10 (IL-10), tumor-associated macrophages (TAMs) are more likely to transition from the anti-tumor M1 phenotype to the pro-tumor M2 phenotype (76). Within the tumor microenvironment (TME), M2 macrophages contribute to tumor promotion by facilitating immune escape (77). Moreover, M2 macrophages can influence the outcomes of radiation, chemotherapy, and immunotherapy by impacting the drug resistance of tumor cells (78). Other immune subsets, such as neutrophils and dendritic cells, also play roles within the TME.

In conclusion, cancer can develop in both individuals with normal insulin levels and those with hyperinsulinemia. However, hyperinsulinemia significantly accelerates cancer progression and alters the tumor microenvironment. This leads to more aggressive cancer phenotypes. These distinctions underscore the importance of understanding the specific mechanisms by which hyperinsulinemia influences cancer to develop targeted interventions.

## 4 Hyperglycemia and cancer progression

**Figure 3:**
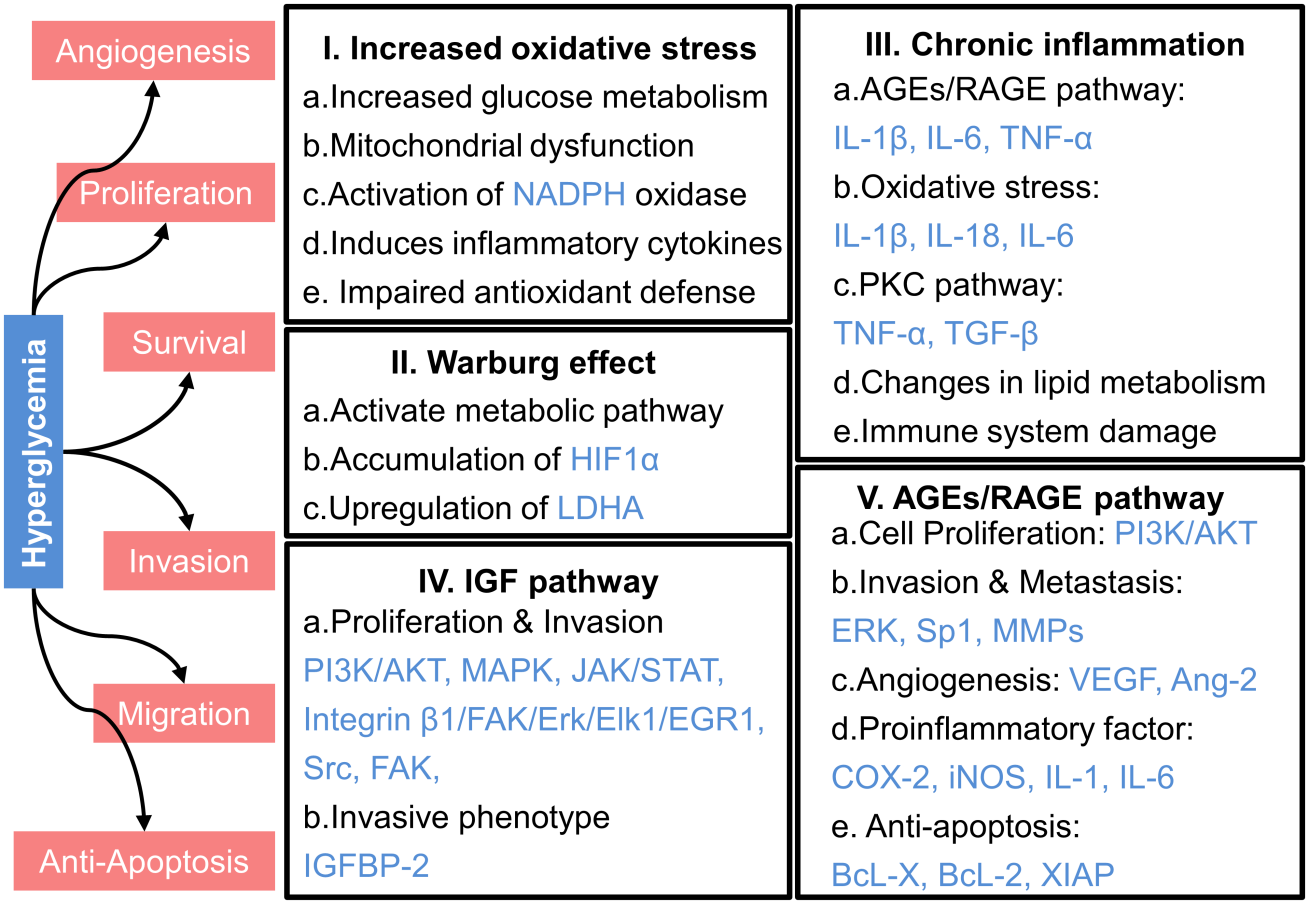
Mechanisms underlying the promotion of cancer progression by hyperglycemia. HIF1α, hypoxia-inducible factor 1α; LDHA, lactate dehydrogenase A; PI3K, phosphatidylinositol 3-kinase; AKT, protein kinase B; MAPK, mitogen-activated protein kinase; JAK, Janus kinase; STAT, signal transducer and activator of transcription; FAK, focal adhesion kinase; Erk, extracellular signal-regulated kinase; Elk1, comprising E26 transformation-specific domain-containing protein Elk-1; EGR1, early growth response protein 1; Src, sarcoma; IGFBP-2, Insulin-like growth factor binding protein-2; IL-1β, interleukin-1β; IL-6, interleukin-6; TNF-α, tumor necrosis factor-α; IL-18, interleukin-18; TGF-β, transforming growth factor-β; Sp1, specificity Protein 1; MMPs, matrix metalloproteinases; VEGF, vascular endothelial growth factor; Ang-2, angiopoietin-2; COX-2, cyclooxygenase-2; iNOS, inducible nitric oxide synthase; BcL-X, B-cell lymphoma-extra large; BcL-2, B-cell lymphoma-2; XIAP, X-linked inhibitor of apoptosis protein.

In patients with T2DM, high blood glucose levels, or hyperglycemia, can influence the development and progression of cancer through various mechanisms, as illustrated in **Figure 3**. These include increased oxidative stress, chronic inflammation, and the activation of specific signaling pathways like the insulin-like growth factor (IGF) and advanced glycation end products (AGEs)/receptor for AGEs (RAGE) pathways.

### 4.1 Increased oxidative stress

Normally, the oxidative environment in the human body maintains a dynamic balance. However, elevated glucose levels in the bloodstream intensify intracellular glucose metabolism, leading to excessive production of reactive oxygen species (ROS). This increased ROS disrupts the electron transport chain, impairs adenosine triphosphate (ATP) synthesis, and promotes mitochondrial dysfunction, further elevating ROS levels (79–81).The accumulation of ROS due to mitochondrial malfunction degrades cellular components and stimulates tumor formation through deoxyribonucleic acid (DNA) mutations and genomic instability (81–83). Moreover, hyperglycemia activates NADPH oxidase, a key ROS generator, thereby exacerbating oxidative stress, damaging DNA, activating oncogenes, and suppressing tumor suppressor genes, all of which contribute to tumor growth and progression (82–84).

Moreover, hyperglycemia induces the production of inflammatory cytokines, such as TNF-α and IL-6 (85, 86), which further elevate ROS levels by activating NADPH oxidase (87). These inflammatory cytokines, along with increased ROS, contribute to chronic inflammation and create a tumor-promoting microenvironment. Normally, antioxidant enzymes neutralize harmful reactive molecules, but hyperglycemia reduces the activity of these enzymes, like catalase and superoxide dismutase (SOD), resulting in ROS accumulation and cellular oxidative damage (88).

Molecular regulation plays a crucial role in the connection between cancer and hyperglycemia-induced oxidative stress. For instance, oxidative stress activates the transcription factor GATA1, which upregulates the expression of von Willebrand Factor, thereby promoting tumor metastasis (89). While the complex interactions between these molecular components are not yet fully understood, it is clear that oxidative stress driven by hyperglycemia can lead to the formation of DNA adducts and chromosomal abnormalities, contributing to genetic instability and cancer development (90, 91).

In conclusion, hyperglycemia impacts tumor development and progression in individuals with T2DM. This effect is more pronounced compared to individuals with normal glucose levels. Hyperglycemia increases oxidative stress, which contributes to cellular damage, genetic mutations, and abnormal cellular development

### 4.2 Warburg effect

The Warburg effect was first discovered by Otto Warburg in the 1950s (92). It describes the tendency of cancer cells to prefer lactate production through glycolysis. This preference occurs even when sufficient oxygen is available for ATP synthesis via oxidative phosphorylation. In normal, well-differentiated cells, energy is primarily generated through a combination of the tricarboxylic acid cycle (TCA) and oxidative phosphorylation, yielding up to 36 mol of ATP from 1 mol of glucose (93). However, in cancer cells, glycolysis predominates even in aerobic conditions, leading to production of lactate rather than ATP (94).

In individuals with normal blood sugar levels, cancer cells may still exhibit the Warburg effect, but this metabolic shift is less pronounced due to the limited availability of glucose. However, in the context of T2DM and associated hyperglycemia, elevated glucose levels significantly amplify the Warburg effect. High glucose levels increase aerobic glycolysis in cancer cells, promoting cancer growth (95, 96). Glucose enters cells via the glucose transporter 1 and triggers various metabolic pathways, some of which increase the invasive potential of cancer cells (97). For instance, high glucose conditions have been shown to upregulate key glycolytic enzymes, such as hexokinase II and pyruvate kinase in breast cancer cells (98) and enolase 1 in gastric cancer cells (99).

In pancreatic cancer, hyperglycemia induces the accumulation of hypoxia-inducible factor 1α, leading to increased lactate dehydrogenase A (LDHA) activity and expression (100, 101). This upregulation elevates the glycolytic rate, accelerating cancer progression and worsening disease outcomes (100, 101). The upregulation of LDHA activity converts pyruvate to lactate, which is extruded through monocarboxylate transporter 4, contributing to the formation of an acidic TME (102). Lactate, once considered a metabolic waste, has intriguingly been discovered to enhance the metabolism of regulatory T cells that infiltrate tumors, allowing cancer cells to evade immune cytotoxicity (103).

In summary, the Warburg effect is a common feature of cancer cells. In individuals with T2DM, hyperglycemia significantly enhances this effect. This leads to increased glycolysis and lactate production. Consequently, the cancer phenotype becomes more aggressive.

### 4.3 Chronic inflammation

Chronic inflammation plays a pivotal role in cancer development and progression through multiple pathways. The chemokines and growth factors generated during the inflammatory response can enhance the development, invasion, and metastasis of cancer cells while preventing normal cell death (104). In individuals with T2DM, hyperglycemia significantly amplifies chronic inflammation compared to normal glucose levels with individuals, accelerating cancer progression (105). In individuals with hyperglycemic, prolonged exposure to high glucose levels increases the formation of AGEs, which interact with the receptor for AGEs (RAGE). This interaction activates several signaling pathways, including NF-κB, MAPK, and Janus kinase/signal transducer and activator of transcription (JAK/STAT), leading to heightened production of inflammatory cytokines like Interleukin-1 β (IL-1β), IL-6, and TNF-α (18). These cytokines exacerbate the inflammatory response, creating a microenvironment that significantly promotes cancer cell proliferation and survival.

Hyperglycemia also increases oxidative stress, leading to the activation of transcription factors such as NF-κB and activator protein-1 (AP-1), and the upregulating genes related to inflammation. This results in the release of inflammatory cytokines like IL-1β, Interleukin-18 (IL-18), and IL-6 (106). The free radicals generated by oxidative stress in hyperglycemic conditions cause more severe DNA damage, contributing to DNA mutations and chromosomal abnormalities underlying cancer development. Moreover, hyperglycemia increases the production of protein kinase C (PKC) isoforms, including PKC-α, PKC-β1, PKC-β2, and PKC-δ (107, 108). The increased activity of PKC can further activate NF-κB and enhance the expression of inflammatory molecules, including TNF-α and TGF-β (109).

Additionally, hyperglycemia disrupts lipid metabolism, causing adipose tissue to produce excess free fatty acids. Elevated free fatty acid levels can induce inflammatory reactions through various signaling pathways, such as the activation of pro-inflammatory serine/threonine protein kinase cascades, which encourage the release of IL-6 and stimulate C-reactive protein production (110). Excess free fatty acids also induce endoplasmic reticulum stress and suppress glucose metabolism, further increasing inflammation (111, 112). Furthermore, high levels of free fatty acids may suppress the expression of genes involved in glucose metabolism, such as glucose transporters, reducing intracellular glucose uptake and increasing the risk of inflammation in hyperglycemic situations (97, 113).

The sustained and intensified inflammatory response in individuals with hyperglycemia not only accelerates cancer cell growth but also impairs the immune system’s ability to detect and eliminate malignant cells (114, 115). This altered immune function, combined with the chronic inflammatory environment, fosters the rapid progression and spread of cancer. On the contrary, normal people have a healthier physiological environment and do not experience long-term inflammatory reactions, which may be one of the reasons for the low risk of cancer. Therefore, a comprehensive approach that includes glycemic control, reduction of oxidative stress, and modulation of inflammatory responses are essential strategies to reduce risk of cancer in patients with T2DM.

### 4.4 The IGF signaling pathway

IGF serves as a critical mediator of growth, development, and survival. It also contributes to an increased risk of cancer. IGF promotes cancer cell proliferation and inhibits apoptosis (116). In normoglycemic individuals, insulin secretion and IGF-1R activation are tightly regulated, resulting in a more controlled rate of cancer cell growth. However, in hyperglycemia individuals, regulation of the IGF signaling pathway influences cancer progression (117). Specifically, elevated glucose levels stimulate the release of more insulin by pancreatic β-cells, leading to higher insulin levels in the bloodstream. This increase in insulin can directly or indirectly impact the IGF-1R, which belongs to the receptor tyrosine kinase family. The activation of IGF-1R by insulin triggers several signaling pathway, including PI3K/AKT, MAPK, JAK/STAT, Sarcoma (Src), and focal adhesion kinase (FAK), all of which collectively enhance cancer cell proliferation, survival, and migration (25). Moreover, insulin may affect the expression of insulin-like growth factor binding protein (IGFBP) to regulate the bioavailability and transport of IGF-1, indirectly affecting its efficacy. For instance, in differentiated 3T3-L1 adipocytes, insulin can stimulate the transcription of the IGFBP-2 gene and increase its secretion (118). Early cancer cell studies have also found that the insulin signaling pathway regulates IGFBP-2 transcription, consistent with the aforementioned results (119). With elevated IGFBP-2 concentrations, the bioavailability of IGF-1 decreases, impeding its transport and potentially weakening its impact on tumor progression. However, epidemiological studies have shown that overexpression of IGFBP-2 is associated with aggressive phenotypes in various human cancers, including glioma, ovarian cancer, prostate cancer, pancreatic cancer, breast cancer, lung cancer, colorectal cancer, melanoma, liver cancer, gastric cancer, rhabdomyosarcoma, and leukemia (120). Exogenous IGFBP-2 can promote the activation of the integrin β1/FAK/ERK/Elk1/EGR1 pathway, thereby stimulating HCC cell proliferation (121). Interestingly, some studies have found that insulin further inhibits the secretion of IGFBP-1 and IGFBP-2 (122), a result in stark contrast to previous findings. Therefore, the action of IGF-1 might be minimally affected by IGFBP but rather influenced by the activation of IGF-1R and its downstream signaling pathways to impact cancer progression.

### 4.5 The AGEs/RAGE signaling pathway

Hyperglycemia leads to the formation of AGEs, which are non-enzymatic glycation products, resulting from the interaction between the aldehyde group of reducing sugars and macromolecules such as proteins, amino acids, lipids, and nucleic acids. In normoglycemic individuals, the formation of AGEs is significantly lower. This reduces the likelihood of AGE accumulation. Consequently, the detrimental effects of AGEs on cellular function are minimized. RAGE, a pattern-recognition receptor in the immunoglobulin superfamily, interacts with extracellular AGEs. RAGE is constitutively expressed in immune cells, lung tissues, and certain cancer cells, including pancreatic cancer (123), non-small cell lung cancer (124), gastric cancer (125), and breast cancer (126). The interaction between AGEs and RAGE activates multiple signaling pathways that critical for tumor growth, angiogenesis, and invasion, such as the PI3K/AKT/mTOR, MAPK, MMPs, vascular endothelial growth factor (VEGF), NF-κB, JAK/STAT, and p53 (127).

In hyperglycemic conditions, the AGE-RAGE interaction is significantly enhanced due to the elevated levels of glucose and subsequent increase in AGE formation. This leads to a more aggressive activation of signaling pathways that promote cancer progression. For example, in PCa-3 cells, the interaction between AGEs and RAGE activates the PI3K/AKT pathway, increasing the phosphorylation of downstream Retinoblastoma protein (Rb) and decreasing overall Rb levels, ultimately enhancing cell proliferation (128). Similarly, hyperglycemia-induced AGE accumulation activates RAGE, leading to elevated ERK phosphorylation and increased expression of MMP-2 and MMP-9, which drive oral cancer cell migrate and further deterioration (129). In gastric cancer, the RAGE/ERK/Sp1/MMP-2 pathway is significantly activated under hyperglycemic conditions, promoting invasion and metastasis (130). Furthermore, the binding of AGEs to RAGE improves transcription of NF-κB and AP-1, resulting in up-regulation of VEGF and ang2 mRNA levels, promoting angiogenesis (131), which plays an important role in the cancer development.

The activation of the AGE/RAGE axis induces a strong pro-inflammatory response. This leads to increased leukocyte activation and apoptosis, accelerated desmoplastic responses, and the recruitment of stromal cells into the TME (132). In hyperglycemic individuals, the chronic elevation of AGEs continuously stimulates RAGE. This exacerbates oxidative stress and promotes NF-κB activation. Consequently, there is an increase in the synthesis and secretion of cytokines, chemokines, and adhesion molecules (133). This hyperactivation contributes to a tumor-promoting microenvironment. It leads to elevated levels of pro-inflammatory cytokines, pro-angiogenic factors, and anti-apoptotic signals. Specifically, the transcription and translation of pro-inflammatory cytokines such as TNF-α, cyclooxygenase-2, inducible nitric oxide synthase, IL-1, and IL-6 are enhanced. Additionally, pro-angiogenic factors and anti-apoptotic signals, including B-cell lymphoma-extra-large (Bcl-xL), Bcl-2, and X-linked inhibitor of apoptosis protein (XIAP), are upregulated (132).

The AGEs/RAGE signaling pathway plays a crucial role in glioma-associated microglia, TAM, and breast cancer-associated fibroblasts (CAFs). The TAM and glioma-associated microglia contribute to tumor development, invasion, and angiogenesis by secreting VEGF and pro-inflammatory cytokines (134). In MDA-MB-231 breast cancer cells, the CAFs promote migration and induce phenotypic alterations linked to invasion by upregulating interleukin-8 (IL-8) levels and activating the IL-8/C-X-C chemokine receptor type 1/2 paracrine signaling pathway (135). Therefore, the AGEs/RAGE signaling pathway can serve as a significant target for cancer therapy. Currently, inhibition of RAGE signaling has effectively suppressed the growth, migration, and invasion of cancer cells (126, 136). In clinical practice, metformin is a commonly used medicine for the treatment of T2DM. Additionally, it has shown therapeutic advantages in those with T2DM and concomitant malignancy. As a result, the combination of metformin and AGE/RAGE inhibitors may be a useful therapeutic approach to treating T2DM-related cancer.

## 5 Hyperlipidemia and cancer progression

**Figure 4:**
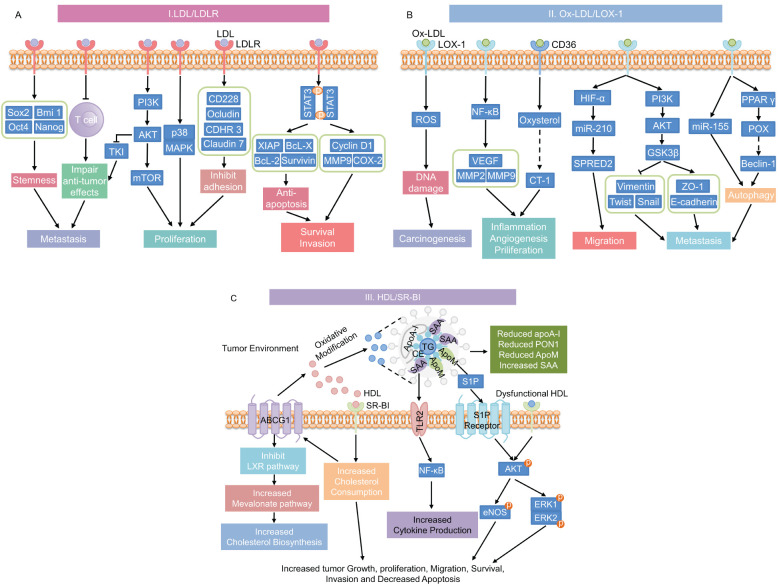
Mechanisms underlying the promotion of cancer progression by hyperlipidemia. **(A)** LDL/LDLR signaling pathway; **(B)** Ox-LDL signaling pathways through its receptors CD36 or LOX-1; and **(C)** HDL and dysfunctional HDL signaling through its receptor SR-BI pathway. This diagram has been modified and pieced together using references (137) and (176) with modifications. LDL, low-density lipoprotein; LDLR, low-density lipoprotein receptor; stemness-related genes includes Sox2, Bmi 1, Oct4, and Nanog; PI3K, phosphatidylinositol 3-kinase; AKT, protein kinase B; TKI, tyrosine kinase inhibit; mTOR, mammalian target of rapamycin; MAPK, mitogen-activated protein kinase; CD228, melanotransferrin; CDHR3, cadherin-related family member 3; STAT3, signal transducer and activator of transcription 3; XIAP, X-linked inhibitor of apoptosis protein; Bcl-x, B-cell lymphoma-extra large; Bcl-2, B-cell lymphoma-2; MMP9, matrix metalloproteinase 9; COX-2, cyclooxygenase-2; Ox-LDL, oxidized LDL; LOX-1, lectin-like oxidized low-density lipoprotein receptor-1; CD36, cluster of differentiation 36; ROS, reactive oxygen species; NF-κB, nuclear factor-κB; VEGF, vascular endothelial growth factor; MMP2, matrix metalloproteinase 2; CT-1, carditorphin 1; HIF-α, hypoxia-inducible factor-α; miR-210, microRNA-210; SPRED2, sprout-related EVH1 domain 2; GSK3β, glycogen synthase kinase 3β; ZO-1, Zonula occludens-1; miR-155, microRNA-210; PPAR γ, Peroxisome proliferator-activated receptor γ; POX, proline oxidase; HDL, high-density lipoprotein; SR-BI, scavenger receptor class B type I; TLR2, toll-like Receptor 2; TG, triglycerides; ApoA-I, Apolipoprotein A-I; SAA, serum amyloid A; CE, Cholesteryl ester; ApoM, Apolipoprotein M; PON1, Paraoxonase-1; S1P, Sphingosine 1-phosphate; eNOS, endothelial nitric oxide synthase; ERK, extracellular signal-regulated kinase.

Dyslipidemia, characterized by increased triglycerides (TG), total cholesterol (TC), and LDL levels, as well as decreased HDL concentrations, plays a complex role in cancer progression (16). The interaction between hyperlipidemia and cancer involves several mechanisms that are not fully understood. The processes through which hyperlipidemia promotes cancer development are shown in **Figure 4**. LDL and its oxidized form, Ox-LDL, promote cancer cell proliferation, angiogenesis, invasion, and metastasis through interactions with receptors such as LDLR, LOX-1, and CD36 (137). Furthermore, HDL, especially when oxidized or glycated in conditions like T2DM, can also enhance cancer cell migration and proliferation (138). These processes are driven by aberrant lipid metabolism and cholesterol accumulation in cancer cells (139). In summary, hyperlipidemia promotes tumor growth in T2DM patients through various mechanisms, which we will further investigate in the following chapters.

### 5.1 Cellular processes

In normal individuals, lipid levels are tightly regulated, preventing excessive intracellular cholesterol, which increases the risk of cancer. However, in hyperlipidemic conditions, abnormal alterations in the TG, TC, LDL, and HDL levels can disrupt cellular signaling pathways, encouraging tumor cell proliferation, invasion, migration, and anti-apoptosis. Interestingly, in prostate cancer, cancer cells can accumulate intracellular cholesterol levels by disrupting normal mitochondria, thereby promoting their own proliferation and migration (140, 141). Additionally, breast, prostate, and pancreatic malignant tumor cells are encouraged to proliferate and invade by LDL cholesterol signaling, which activates the AKT, ERK, and STAT3 pathways (142, 143). The LDLR superfamily member low-density lipoprotein receptor-related protein 1 promotes tumor cell motility and invasion by modulating MMP-2 and MMP-9 production. It also suppresses cell apoptosis by modulating IR, serine/threonine protein kinase signaling pathways, and cysteine-aspartic acid protease-3 expression (15).

### 5.2 Tumor microenvironment, angiogenesis and immunosuppression

The tumor microenvironment (TME) is a dynamic system of cells, chemicals, and stromal components that support tumor growth, invasion, and metastasis. In hyperlipidemic conditions, elevated lipid levels further exacerbate these processes compared to individuals with normal lipid levels. Angiogenesis, a critical factor in tumor progression, is particularly enhanced under hypoxic conditions. HDL contributes to this by stimulating the PI3K/Akt pathway via the scavenger receptor class B-I (SR-BI), leading to the accumulation of hypoxia-inducible factor-1α (HIF-1α) and the activation of angiogenic protein transcription (144, 145). Additionally, HDL increases the phosphorylation of the vascular endothelial growth factor receptor 2, which in turn activates the downstream pathways ERK1/2 and p38 MAPK to promote angiogenesis (146). However, HDL can also modulate macrophages within the inflammatory TME, potentially inhibiting pathological angiogenesis (146).

Cholesterol plays a pivotal role in modulating the immune response within the TME. It activates the ER stress sensor X-box binding protein 1 (XBP1) in CD8+ T cells, leading to the transcription of proteins like programmed cell death protein-1 (PD-1) and CD244, contributing to T cell exhaustion (147). Cholesterol efflux in TAMs also enhances their tumor-promoting properties by increasing IL-4 signaling and reducing IFN-γ-induced gene expression (148).

### 5.3 The LDL and Ox-LDL signal pathways

LDL is a key plasma lipoprotein that primarily transports cholesterol throughout the body. Upon oxidation, LDL forms Ox-LDL (149), which plays a significant role in various pathological conditions, including cancer progression. In normal individuals, where blood lipid levels remain in a dynamic balance, LDL and Ox-LDL signaling contribute to a low risk of cancer. However, in individuals with hyperlipidemia, elevated LDL levels significantly alter cancer progression dynamics, leading to more aggressive tumor growth and resistance to therapy.

Normally, LDL binds to LDLR on the cell surface, where it is internalized and processed to release cholesterol for cellular use (150). This process is tightly regulated by mechanisms involving sterol regulatory element-binding protein-2 (SREBP-2), SREBP cleavage-activating protein, and proprotein convertase subtilisin/kexin 9 (151). However, cancer cells can exploit this pathway by upregulating LDLR expression, leading to increased cholesterol uptake, which supports rapid cell proliferation and tumor growth (152). Although this mechanism is present in normolipidemic individuals, its impact is amplified in hyperlipidemic conditions. In such cases, excess LDL supplies a larger amount of cholesterol and lipids for energy, further fueling cancer cell metabolism. Dysregulated lipid metabolism can result in lipotoxicity and elevated oxidative stress, which enhances the susceptibility of LDL to oxidation, forming Ox-LDL (137, 153). Ox-LDL binds to receptors such as LOX-1 and CD36, triggering cascades of oncogenic signals that are more pronounced in hyperlipidemic conditions. This leads to mutations, promotion the epithelium-mesenchyme transition, initiation protective autophagy, and production of growth factors, cytokines, and pro-inflammatory markers. The resulting increase in ROS and pro-inflammatory markers significantly contributes to cancer progression and resistance to chemotherapy (137).

Research into the connection between LDL and Ox-LDL and their effects on cancer is still in its early stages. In MDA-MB-231 breast cancer cells, an increase in LDL levels (greater than 1.5 times that of the control group) led to upregulation of 147 mapped genes (including pERK, pAKT, and pJNK) and downregulation of 95 mapped genes (including CD226, Claudin7, Ocludin, and integrinβ8), while reducing cell adhesion and promoting cell migration and proliferation (142, 154–157). LDL activates STAT3 and JAK1, JAK2, and Src in prostate and pancreatic tumor cells, promoting cancer cell proliferation, migration, invasion, and the up-regulation of numerous oncogene products (143). Furthermore, increased LDL cholesterol in kidney carcinoma activates the PI3K/AKT signaling pathway, which inhibits the anticancer effects of tyrosine kinase inhibitors (158). It’s important to note that cancer cells may be able to evade immune surveillance at high LDL levels. When V9γδ2 T cells are activated and express LDLR, uptake of low-density lipoprotein cholesterol leads to decreased mitochondrial mass and reduced ATP production, resulting in inhibition of the antitumor function of V9γδ2 T cells (159).

Ox-LDL has been demonstrated to induce mutagenesis and enhance cancer cell proliferation, migration, invasion, and treatment resistance, in addition to the impact of LDL on cancer cells (137, 160). In an experimental investigation on primary rat hepatocytes, Ox-LDL, the principal component of 4-hydroxynonenal and lipotoxic, encouraged micronucleus formation, chromosomal aberration, and increased sister chromatid exchange frequency at concentrations ranging from 0.1-10 μM, resulting in enhanced DNA damage and induced mutation (161). Similarly, up-regulation of microRNA-210 and HIF-1 by Ox-LDL increases the risk of vascular disease and cancer (162, 163). Furthermore, Ox-LDL receptors such as LOX-1 and CD36, along with associated downstream signaling cascades, play an important role in cancer progression. Recent investigations have demonstrated that the Ox-LDL/LOX-1 axis facilitates the migration of cancer cells by attracting neutrophils to tumor endothelial cells (164). Additionally, the interaction of Ox-LDL with LOX-1 activates NF-κB target genes like VEGF, MMP-2, and MMP-9 to encourage the growth, invasion, and angiogenesis of cancer cells (137, 160). In prostate cancer cells, Ox-LOL-induced overexpression of LOX-1 resulted in epithelial-mesenchymal transformation and promoted cancer cell invasion and migration by reducing the expression of epithelial markers (such as cadherin and platelet globin) and increasing the expression of mesenchymal markers (like vimentin, N-cadherin, snails, slugs, MMP-2, and MMP-9) (165).

CD36 is another Ox-LDL receptor expressed in various cell types, including monocyte macrophages, microvascular endothelial cells, dendritic cells, and tumor cells. CD36 primarily regulates cellular lipid metabolism but also mediates lipid uptake, immune recognition, inflammation, molecular adhesion, and apoptosis (166, 167). CD36-mediated Ox-LDL uptake causes CD8+ T cell lipid peroxidation, which inhibits IFN-γ and TNF production via p38 kinase activation, favoring cancer cell proliferation (166). Ox-LDL increase the association of CD36 with JAK2, resulting in increased bladder cancer dryness by promoting JAK2 phosphorylation and activating the STAT3 signaling cascade (168). Neurite outgrowth inhibitor-B (Nogo-B) expression is directly upregulated by CD36-mediated Ox-LDL uptake. Nogo-B interacts with autophagy-related 5 (ATG5) to promote autophagy. These process leads to lysophosphatidic acid-enhanced yes-associated protein carcinogenic activity (169). Another significant mechanism through which Ox-LDL promotes cancer progression is autophagy. Research indicates that Ox-LDL can activate the critical metabolic enzyme OPLINe oxidase, increasing autophagy in cancer cells via pathways involving Ox-LDL and peroxisome proliferator-activated receptor (137).

In conclusion, LDL and Ox-LDL signaling pathways contribute to cancer progression in both normolipidemic and hyperlipidemic individuals. However, the effects are significantly more aggressive in those with hyperlipidemia. Elevated levels of LDL and Ox-LDL in hyperlipidemic individuals lead to increased cancer cell proliferation, invasion, and resistance to therapy. This makes hyperlipidemia a critical factor in cancer development. Therefore, lowering LDL and Ox-LDL levels could be a promising therapeutic strategy for preventing and managing cancer, particularly in individuals with T2DM.

### 5.4 The HDL signal pathways

HDL, often referred to as “good” cholesterol, plays a critical role in maintaining lipid homeostasis and has been shown to exert anti-inflammatory, anti-oxidative, and anti-tumor effects in the TME (170–172). In individuals with normal lipid levels, HDL functions optimally. It facilitates reverse cholesterol transport (173), reducing oxidative stress, and mitigating inflammation. Together, these actions contribute to a reduced risk of cancer progression (171, 172). However, in T2DM patients, HDL undergoes structural and compositional changes, resulting in dysfunctional lipoproteins (174, 175). Elevated cholesteryl ester transfer protein (CETP) activity and reduced lecithin cholesterol acyltransferase (LCAT) activity lead to altered in HDL particle size and composition, resulting in dysfunctional HDL. This dysfunction is marked by a decrease in key proteins such as apolipoprotein A-I and paraoxonase 1, along with an increase in triglycerides and serum amyloid A (SAA) (176, 177). These alterations negatively impact HDL’s ability to counteract oxidative stress and inflammation, crucial mechanisms that normally accelerate cancer development.

The accumulation of SAA in HDL particles enhances its binding to Toll-like receptor 2 (TLR2), triggering the NF-κB signaling pathway, which promotes production of pro-inflammatory cytokines. This pro-inflammatory state creates a conducive environment for cancer initiation and progression, contrasting with the protective anti-inflammatory role of HDL in individuals with normal lipid profiles (178, 179). Additionally, lower levels of apolipoprotein M in HDL lead to elevated free sphingosine-1-phosphate (S1P) levels, which further exacerbate tumor growth, migration, and angiogenesis through the AKT phosphorylation pathway (176). Moreover, the glycation and oxidation of HDL, particularly prevalent in T2DM patients, have been implicated in the progression of cancers such as breast cancer (180). Oxidized and glycated HDL affects MDA-MB-231 breast cancer cells through the AKT/ERK signaling pathway, increasing integrin expression and enhancing cancer cell growth, migration, and invasion (181). Additionally, through enhancing lipid internalization and cholesterol intake, SR-BI overexpression in cancer cells encourages cell growth and proliferation (182, 183).

In conclusion, HDL generally protects against cancer in individuals with normal lipid levels. However, in hyperlipidemic conditions, its functionality is compromised, leading to a higher risk of cancer development. These differences in HDL signaling pathways highlight the importance of managing lipid levels to reduce cancer risks, especially in T2DM patients.

## 6 Other factors and cancer progression

The connection between T2DM and cancer is significantly influenced by the gut microbiome, a rapidly emerging area of research. The gut microbiota, consisting 500–1000 species and 10^14^ bacteria—ten times more abundant than human cells—acts as a complex endocrine organ. It plays vital roles in digestion, nutrient absorption, and reinforcement of the intestinal immune system. Additionally, it maintains the intestinal mucosal barrier, preventing harmful substance infiltration, synthesizes beneficial compounds like vitamins, regulates host metabolism, and reduces cancer risk while enhancing anti-tumor responses (184–190).

Studies have demonstrated a strong connection between T2DM and the gut microbiota. Research by Larsen in 2010 revealed significant changes in gut microbial composition between individuals with and without T2DM (191). Ma’s subsequent summary highlighted a reduction in beneficial bacteria and an increase in harmful and potentially pathogenic bacteria in T2DM patients (184). Dysbiosis, or an imbalance in the gut microbiota, has been associated with various illnesses, including diabetes, metabolic syndrome, non-alcoholic fatty liver disease, and even mental disorders such as depression and multiple sclerosis (192). Moreover, abnormal gut microbiota changes have been linked to a higher risk of several cancers, including colorectal cancer (193), hepatocellular carcinoma (194), non-small cell lung cancer (195), and prostate cancer (196).

The gut microbiota plays a crucial role in human health and disease development. It influences immune cell activity and cancer risk through its remarkable metabolic abilities (197). Through the metabolic byproducts it produces, the gut microbiota may be able to indirectly cause the development of cancer in distant organs such as the pancreas, liver, breast, lung, prostate, and stomach (198). The primary metabolic products of the gut microbiota are short-chain fatty acids (SCFAs). Historically, SCFAs have been primarily studied for their anti-inflammatory properties. Recent research, however, has uncovered a unique carcinogenic pathway called the “gut-prostate axis”, where SCFAs produced by specific bacteria (e.g., Rikenellaceae, Alistipes, and Lachnospira) promote PCa growth (199). Additionally, the gut microbiota indirectly impacts PCa progression through other metabolic products such as testosterone, estrogen, folate, and phenylacetylglutamine (196).

The gut microbiota also influences tumor immune responses through various mechanisms, including stimulating regulatory T cell proliferation, inducing IgA expression, regulating antimicrobial peptides and systemic inflammation, and affecting bacterial translocation (196). The intestinal mucosal barrier, composed of tightly bound epithelial cells, usually separates the gut microbiota from immune cells. However, specific changes in gut microbial composition can stimulate the mucosal immune system, leading to chronic inflammation and mucosal damage. This imbalance in intestinal mucosal immunity may cause cellular and DNA damage, genetic mutations, activation of tumor-associated signaling pathways, and ultimately contribute to tumor development and progression (197, 200).

The relationship between gut microbiota, T2DM, and cancer is complex and remains an active area of research. While current data suggest a strong connection, there is insufficient evidence to definitively conclude that gut microbiota dysbiosis directly causes cancer in T2DM patients or to fully elucidate the specific mechanisms involved. Therefore, further research is necessary to better understand these interactions and underlying mechanisms.

## 7 Conclusion

In normal individuals, cancer development is typically driven by genetic factors, environmental exposures (such as smoking or radiation), and chronic inflammation. These individuals usually maintain balanced metabolic states, including normal blood sugar, insulin, and lipid levels. However, in T2DM patients, cancer progression is accelerated due to hyperglycemia, hyperinsulinemia, and hyperlipidemia. Hyperglycemia increases oxidative stress and chronic inflammation, leading to DNA damage and promoting the Warburg effect, which fuels cancer cell metabolism. Hyperinsulinemia activates critical signaling pathways, such as PI3K/AKT, mTOR, and Ras/MAPK, which drive cell proliferation, survival, and resistance to apoptosis. Additionally, hyperinsulinemia lowers SHBG levels, increasing the availability of sex hormones like estrogen, thereby heightening the risk of hormone-dependent cancers. Hyperlipidemia contributes by altering lipid metabolism, influencing cellular signaling within the tumor microenvironment, and promoting angiogenesis and immune suppression, all of which support tumor growth and metastasis.

These metabolic abnormalities do not act in isolation but are interrelated, creating a pro-tumorigenic environment through synergistic interactions. Hyperinsulinemia and hyperglycemia, often exacerbated by T2DM, induce insulin resistance and elevate IGF-1 levels, further activating the PI3K/AKT pathway and supporting tumor cell proliferation. Hyperglycemia and hyperlipidemia provide essential metabolic substrates that fuel cancer cell glycolysis and lipid synthesis, accelerating tumor growth. Moreover, the chronic inflammation and oxidative stress associated with these conditions activate pro-inflammatory pathways like NF-κB and transcription factors such as HIF-1α, enhancing tumor angiogenesis, metastasis, and overall progression.

In summary, the metabolic abnormalities in T2DM significantly accelerate cancer progression. Understanding the complex interplay between these metabolic states and cancer is crucial for developing targeted interventions. Future research should focus on elucidating the precise molecular mechanisms underlying these interactions and exploring the potential of insulin sensitizers, metabolic modulators, and anti-inflammatory agents as therapeutic strategies. Personalized approaches that integrate metabolic management with conventional cancer therapies may offer improved treatment efficacy and outcomes for patients with T2DM-related malignancies. Continued investigation into these areas could pave the way for novel therapies that address both metabolic disorders and cancer progression.
